# The Plasma Levels of 3-Hydroxybutyrate, Dityrosine, and Other Markers of Oxidative Stress and Energy Metabolism in Major Depressive Disorder

**DOI:** 10.3390/diagnostics12040813

**Published:** 2022-03-26

**Authors:** Michaela Krivosova, Eduard Gondas, Radovan Murin, Matus Dohal, Igor Ondrejka, Ingrid Tonhajzerova, Peter Hutka, Nikola Ferencova, Zuzana Visnovcova, Igor Hrtanek, Juraj Mokry

**Affiliations:** 1 Biomedical Centre Martin, Jessenius Faculty of Medicine in Martin, Comenius University in Bratislava, 03601 Martin, Slovakia; michaela.krivosova@uniba.sk (M.K.); nikola.ferencova@uniba.sk (N.F.); zuzana.visnovcova@uniba.sk (Z.V.); 2 Department of Medical Biochemistry, Jessenius Faculty of Medicine in Martin, Comenius University in Bratislava, 03601 Martin, Slovakia; gondas3@uniba.sk (E.G.); radovan.murin@uniba.sk (R.M.); 3 Department of Pharmacology, Jessenius Faculty of Medicine in Martin, Comenius University in Bratislava, 03601 Martin, Slovakia; dohal1@uniba.sk; 4 Psychiatric Clinic, Jessenius Faculty of Medicine in Martin, Comenius University in Bratislava, University Hospital Martin, 03659 Martin, Slovakia; igor.ondrejka@uniba.sk (I.O.); hutka6@uniba.sk (P.H.); igor.hrtanek@uniba.sk (I.H.); 5 Department of Physiology, Jessenius Faculty of Medicine in Martin, Comenius University in Bratislava, 03601 Martin, Slovakia; ingrid.tonhajzerova@uniba.sk

**Keywords:** 3-hydroxybutyrate, NADH, dityrosine, myeloperoxidase, major depressive disorder, energy metabolism, oxidative stress

## Abstract

Major depressive disorder (MDD) is a serious mental disease with a pathophysiology that is not yet fully clarified. An increasing number of studies show an association of MDD with energy metabolism alteration and the presence of oxidative stress. We aimed to evaluate plasma levels of 3-hydroxybutyrate (3HB), NADH, myeloperoxidase, and dityrosine (di-Tyr) in adolescent and adult patients with MDD, compare them with healthy age-matched controls, and assess the effect of antidepressant treatment during hospitalisation on these levels. In our study, plasmatic levels of 3HB were elevated in both adolescents (by 55%; *p* = 0.0004) and adults (by 88%; *p* < 0.0001) with MDD compared to controls. Levels of dityrosine were increased in MDD adults (by 19%; *p* = 0.0092) but not adolescents. We have not found any significant effect of antidepressants on the selected parameters during the short observation period. Our study supports the findings suggesting altered energy metabolism in MDD and demonstrates its presence independently of the age of the patients.

## 1. Introduction

Major depressive disorder (MDD) is a mood disorder with a high lifetime prevalence, particularly in some European countries [1]. The pathophysiology of MDD has not yet been fully clarified, although various mechanisms such as altered neurotransmission, dysregulation of the hypothalamus–pituitary–adrenal axis, reduced hippocampal volume, increased systemic inflammation, and influence of genetic factors have been described [2,3]. Even though onset often begins in childhood, different pathophysiological mechanisms might be present in adolescents and adults with MDD [4]. One of the current research strategies is a robust identification of biomarkers useful for diagnosis, severity and prognosis determination, and antidepressant response monitoring. A recent systematic review on metabolomics in MDD and bipolar diseases (BD) identified three possible metabolic pathways involved in the pathophysiology of these diseases—energy/mitochondrial metabolism, glutamatergic metabolism, and neurotransmission [5]. Energy metabolism imbalances due to impaired oxidative phosphorylation have a role in the etiology of neurological diseases. Such alterations are linked with oxidative stress (OS) [6] and a positive association between OS and depressive disorder has been already discussed [7,8,9,10]. The oxidative stress hypothesis of depressive disorder was proposed suggesting volumetric reductions in specific brain areas, such as the prefrontal cortex (PFC) and hippocampus, could be the causes of elevated OS and thus increased free radical production in these regions [9,11].

One of the putative OS markers could be *o*,*o*′-dityrosine (di-Tyr). It is metabolically stable and excreted as non-changed in urine, enabling easy detection both in plasma and urine. In conditions of whole-body oxidative stress, such as atherosclerosis, acute inflammation, or systemic bacterial infections, di-Tyr has been shown to be a potential biomarker as its concentrations were significantly elevated compared to levels of healthy individuals. It is formed after oxidative stress in a protein leading to subsequent proteolysis. Hence, it is considered not only a marker for oxidative protein damage, but it may also serve as a “marking” step for protein degradation [12,13]. Myeloperoxidase is an enzyme that catalyses the production of free radical derivatives and contributes to the oxidation of L-Tyr to a stable di-Tyr. Apart from immune cells, it is also expressed in the brain, where its increased expression was related to neurodegeneration [14,15].

Energy metabolism and cellular reducing potential/status could be reflected by NAD(P)H to NAD(P)^+^ ratio. Nicotinamide adenine dinucleotide (NADH) acts as a carrier of electrons that can donate to the respiratory chain. Depressed patients were found to have reduced blood flow and glucose metabolism in the specific brain areas such as PFC [16]. Several metabolic processes could affect the cytosolic ratio of NADH/NAD^+^ and subsequently could exert their effect on glycolytic glucose flow and blood flow rate in the brain [17,18]. Nicotinamide adenine dinucleotide phosphate (NADPH) is another carrier of electrons with roles in cellular anabolic metabolism of lipids and antioxidative protection. It is an indispensable substrate for maintaining and regeneration of glutathione and cytoplasmic coenzyme Q10 and thus contributes to antioxidant defence [19,20]. The metabolism of NADPH is linked with several metabolic pathways, including energy metabolism [21].

3-hydroxybutyrate (3HB), also known as β-hydroxybutyrate, is one of the ketone bodies and supports mammalian survival by serving as an alternative source of ATP during conditions with energy deficits. Its higher levels were measured during starvation, caloric restriction, high-intensity exercise, and a low-carbohydrate ketogenic diet [22,23]. 3HB is an essential source of acetyl moieties for sustaining the cellular metabolism in the brain. In addition to its metabolic function, 3HB possesses the signalling and regulatory roles through receptors and histone methylation [24]. One of the 3HB receptors, hydroxycarboxylic acid receptor 2 (HCAR2), has been highly expressed in macrophages and microglia in the brain showing anti-inflammatory and neuroprotective effects [25,26]. 3HB itself has been proposed to control emotional systems in the brain through energy metabolism processes [27]. It has been shown that mild elevation of ketone bodies (3HB, acetoacetate) in the blood occurs physiologically during the process of aging [28]. Recently, serum 3HB concentrations have been proposed as markers of depression severity [27,29].

To evaluate oxidative damage, levels of oxidative stress products or levels of antioxidant enzymes and molecules might be estimated. This experimental study aimed to identify any abnormalities regarding energy metabolism and oxidative stress from peripheral blood in adolescent and adult patients with MDD and observe the effect of a short antidepressant treatment.

## 2. Materials and Methods

### 2.1. Population and Blood Samples

This study followed the Declaration of Helsinki, approval was gained by the Ethics Committee of the Jessenius Faculty of Medicine, Comenius University in Bratislava (No EK 1983/2017), and all study subjects signed the written consent. The consent of parents was given in the case of underage patients. Adolescent and adult inpatients diagnosed with MDD from the Psychiatric Clinic of Jessenius Faculty of Medicine in Martin, Comenius University in Bratislava, and University Hospital Martin, Slovakia, were included in the study. The diagnosis of MDD without psychotic symptoms was assessed by detailed clinical examination using unstructured diagnostic interview by either a child/adolescent psychiatrist or an adult psychiatrist according to the Diagnostic and Statistical Manual of Mental Disorders, DSM-5 [30]. The Clinical Global Impression rating scale was used for the assessment of depression severity [31]. 

During the hospital stay, adolescent patients were taking fluoxetine (15–40 mg) or vortioxetine (10–15 mg); adult patients were taking duloxetine (30–120 mg), escitalopram (10–20 mg), venlafaxine (225–300 mg), vortioxetine (20 mg), or a combination as directed by their doctor. Patients’ peripheral blood samples were collected at hospital admission (case1) and after a certain period of antidepressant treatment (case2; in adolescents on seventh day of hospitalisation and in adult patients at discharge day—10–27 days; on average, on the 21st day). The children’s first blood sample was collected before their meal and morning medication and the second sample two weeks under the same conditions. Adult samples were both withdrawn after night-fasting. Age-matched volunteers were included in the study as healthy controls and their fasting blood samples were withdrawn in the morning hours. All blood samples were collected in EDTA tubes and shortly after the withdrawal (within 2 h), the plasma was obtained by centrifugation (4 °C, 15 min, 590 g) and stored at −80 °C until the analysis.

### 2.2. Enzymatic Estimation of 3-Hydroxybutyrate Level in Plasma

The concentration of 3-hydroxybutyrate in plasma was determined enzymatically by modifying the previously published method [32]. Briefly, 30 µL of plasma was mixed with 270 µL of the reaction mixture (0.2 M glycine/0.16 M hydrazine buffer pH 9.0 supplemented with 5 mM NAD+) in a 96-well plate. After reading the initial absorbance at 340 nm (A0) by SynergyTM plate reader (Bio Tek, Winooski, VT, USA), 3-hydroxybutyrate dehydrogenase was added to the final activity 50 mU/well. After incubation at 37 °C for 60 min, the end-point absorbance at 340 nm (AE) was measured. The concentration of 3-hydroxybutyrate in plasma was estimated based on the calculated amount of generated NADH, using the value of extinction coefficient for NADH equal to 6.22 mM^−1^·cm^−1^.

### 2.3. Estimation of Relative Levels of Dityrosine, NADH and Tryptophan in Plasma

The relative levels of dityrosine [33], NADH [34] and tryptophan [35] were determined by measuring their natural fluorescence. Plasma (5 µL) was mixed with 250 µL of glycine/hydrazine buffer pH 9.0 and the intensity of dityrosine, NADH and tryptophan fluorescence was measured by SynergyTM plate reader (Bio Tek, USA). The pairs of emission and extinction wavelength to estimate the fluorescence intensity of dityrosine, NADH or tryptophan were 325 nm/410 ± 20 nm, 340 nm/440 ± 20, or 280 nm/348 ± 9 nm, respectively. The intensity of natural fluorescence of dityrosine, NADH or tryptophan estimated in plasma obtained from healthy individuals was considered as 100%. Levels of tryptophan were measured only for protein level standardization.

### 2.4. Levels of Myeloperoxidase in Plasma

The plasmatic concentration of myeloperoxidase was measured using a commercial enzyme-linked immunosorbent assay (ELISA) kit (Abcam, Cambridge, UK) with the concentration range 312–20,000 pg/mL and sensitivity of 10 pg/mL. The pilot study was performed to estimate the dilution factor for plasma samples. The samples were run in singlets with no dilution. The analysis was performed according to the manufacturer´s protocol and the final absorbance was measured at 450 nm using multimode plate reader Varioskan (Thermo Fisher Scientific, Waltham, MA, USA). The concentration was calculated using four parametric logistic curves in an online calculator (MyAssays, Brighton, UK).

### 2.5. Statistical Methods

The statistical analyses were performed in GraphPad Prism 8.0.1 (GraphPad, San Diego, CA, USA). The data were tested for normality by a Shapiro–Wilk test, and outliers were identified and excluded using the ROUT test with Q = 1%. Accordingly, a parametric paired *t*-test (normally distributed data) or Wilcoxon matched test (not normally distributed) was used to compare levels in case1 and case2 groups, while parametric unpaired *t*-test (normally distributed data) or a Mann–Whitney unpaired test (not normally distributed) were used to compare patients (case1) and controls. A *p*-value < 0.05 was considered statistically significant. The impact of age and duration of hospitalisation was analysed using linear regression and correlation. The impact of gender was measured by comparing female and male groups with an unpaired *t*-test or nonparametric tests. Power analysis (effect size, power) was performed post-hoc using G*Power 3.1.9.4 (Dusseldorf University, Dusseldorf, Germany) with the following input parameters: difference between two (in)dependent means, two tails, α = 0.05, and sample size according to the real group size.

## 3. Results

In our study, there were 11 depressed adolescents (15–18 years old) with 20 age-matched controls (16–18 years old) and 21 depressed adult patients (52–75 years old) with 15 age-matched controls (50–77 years old). For the assessment of the influence of antidepressant therapy on the levels of observed parameters, we used 11 adolescent samples but only 15 elderly patients’ samples (case2). The basic demographic data are displayed in Table 1.

**Figure 1:**
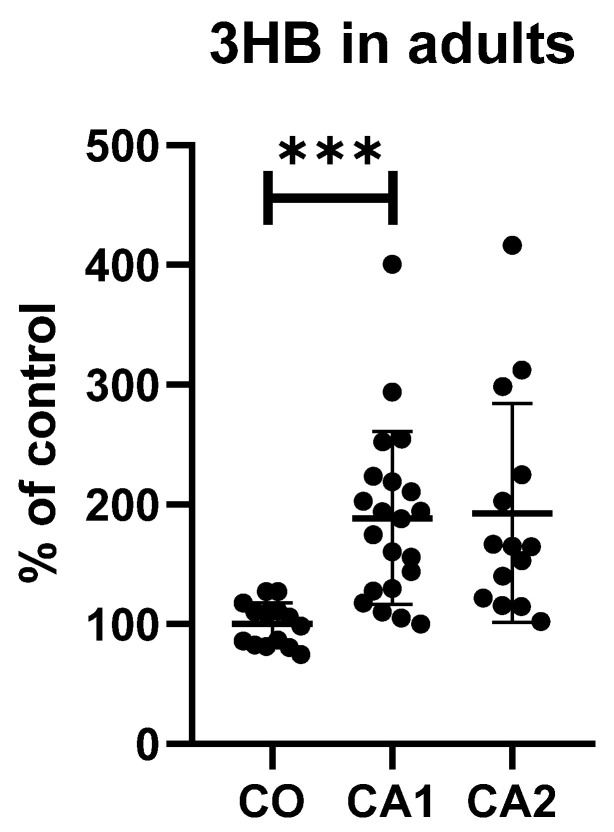
Levels of 3HB in adult patients and controls. CA1—case1, CA2—case2, CO—controls; *** *p* < 0.001.

**Figure 2:**
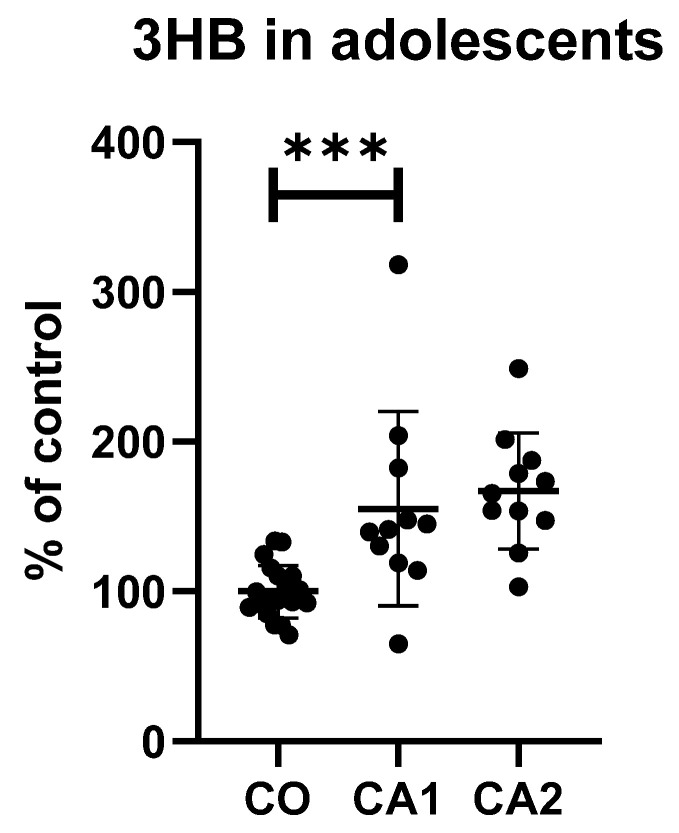
Levels of 3HB in adolescent patients and controls. CA1—case1, CA2—case2, CO—controls; *** *p* < 0.001.

All of the results between depressed patients and controls and after a short antidepressant treatment during hospitalisation are shown in Table 2. Moreover, a comparison of 3HB levels in adolescents and adult subjects are in Figure 1 and Figure 2.

The levels of 3HB were elevated by 55% and 89% in adolescents and adults with MDD compared to healthy controls, respectively. Dityrosine levels were increased by 19% in adults with MDD compared to controls. The achieved power of the measurements with statistically significant differences was the following: in the adult group, 0.83 and 1.00 for di-Tyr and 3HB, respectively (effect sizes were 1.04 and 1.68); in the adolescent group, 0.85 for 3HB (effect size d = 1.17).

We have not found that age impacted the levels of measured parameters in adolescents. However, male adolescent controls had significantly higher levels of NADH (0.40 ± 0.05) compared to female controls (0.35 ± 0.04; *p* = 0.0279). Linear regression analysis showed the negative effect of age on 3HB levels in samples from adult patients. A low number of samples in the female adult control group limited the gender correlation within that group. We have not found any significant correlation between plasmatic levels of 3HB and duration of hospital stay in MDD adult patients (*p* = 0.3891).

## 4. Discussion

The present study assessed the selected parameters related to energy metabolism and oxidative stress in adolescent and adult patients with MDD. The parameters were chosen in line with the growing evidence of altered ATP production and electron transport chains, and mitochondrial dysfunction in MDD [36,37]. Along with these studies, even the effect of ketamine, a rapid antidepressant, was recently suggested to be mediated by mitochondrial energy metabolism and antioxidant defence system [38]. We aimed to measure these parameters in two different age groups—adolescents and older adults to compare the possible pathophysiological mechanism difference related to the age of MDD patients. We chose to quantify the level of myeloperoxidase, an enzyme involved in oxidative stress, and interrelated dityrosine levels. Moreover, by measuring NADH and 3HB in plasma, we could partially evaluate the peripheral conditions associated with redox potential and energy metabolism in MDD patients.

The concentration of 3HB was measured in all the study subjects higher than the reference range of 0.01–0.25 mmol/L in non-fasting plasma samples [39]. This might be explained by the overnight fasting of controls and patients at hospital admission. However, we found that the plasmatic levels of 3HB were more elevated in patients with MDD compared to healthy age-matched subjects, which was observed in both studied age groups of depressed individuals. We confirmed that increased 3HB levels are present independently of the age of the patients during an acute depressive episode and possibly also chronically, as we did not observe any decrease after proper antidepressant therapy during hospitalisation.

The explanation of elevated plasmatic ketone bodies in MDD might lie in a higher demand in energy utilization and/or insulin insensitivity in patients with major depression [40,41]. However, elevated 3HB levels were also found in the serum of patients with schizophrenia compared to healthy subjects [40,42], which suggests that this marker could generally reflect psychosomatic stress [29]. There was also a previously reported correlation between high 3HB levels in serum and effectiveness of antidepressant therapy with sertraline or venlafaxine where higher 3HB levels were associated with symptoms resolution [29]. In our study, the parameter reflecting effectiveness of antidepressant therapy was the duration of the hospitalisation of patients. However, the second blood samples from adolescents were withdrawn after too short of a time period (1 week), and in the group of adult inpatients, no significant correlation was discovered.

Stress itself creates the conditions for adipocyte lipolysis and consequential long-chain fatty acid degradation to ketone bodies. A study by Kubera et al. [43] showed that psychosocial stress elevated serum 3HB concentrations in normal-weight men implying extra energy demand to satisfy its increased needs. Considering insulin insensitivity, pro-inflammatory cytokines associated with desensitised glucocorticoid receptors are of great importance. Due to chronic stress, levels of glucocorticoids are increased and inhibit the expression of GLUT4 transporter. Moreover, the cortisol-induced release of fatty acids from lipoproteins further alters insulin receptor signalling and increases pro-inflammatory cytokines, e.g., tumour necrosis factor (TNF)-α contributes to insulin desensitisation [41,44,45]. 

Apart from 3HB’s role as a metabolism intermediate, it possesses regulatory functions influencing gene expression, neuronal protection, overall metabolic rate, and it is an important epigenetic regulator by inhibiting histone deacetylases [24]. A recent experimental study showed the anti-inflammatory effect of 3HB in hippocampus of rats exposed to acute and chronic stress. Hippocampal levels of TNFα and IL-1β were reduced after 3HB administration, which was likely mediated by the inhibition of the NLRP3 inflammasome [46]. Similar results were found in the levels of IL-1β and IL-18 in human monocytes [23].

Regarding the finding that levels of 3HB decreased with the age of adult patients, it could be caused by age-related reduced lipolytic activity or reduced ketogenic activity due to impaired mitochondrial function [47].

The biosynthesis of 3HB from acetoacetate is associated with NAD^+^ regeneration from reduced-form NADH [24]. Although NAD^+^ is predominantly an intracellular nucleotide, it also plays important extracellular roles [48,49]. The oxidized and reduced form NAD^+^/NADH ratio in plasma might provide essential information regarding redox metabolism disorders or cellular bioenergetics alterations. Physiologically, increased levels of reduced NAD^+^ are also reported in relation to aging [50], but we have not found any correlation. 

In our study, we measured only levels of NADH in plasma. We have not observed any significant difference between the levels of depressed patients and healthy controls, nor after the short antidepressant treatment. To the best of our knowledge, the levels of NADH, NAD^+,^ or their ratio in the plasma of MDD patients have not yet been characterized yet. Increased levels of NADH in plasma with decreased NAD^+^ and NAD^+^/NADH ratio were previously found in children with autism [51], in cerebrospinal fluid in neurodegenerative diseases were levels of NAD^+^ increased, but no change was observed in plasma [52]. Nonetheless, we found increased levels of NADH in female adolescents compared to male adolescents, but not in adults. The same was detected in a recent study suggesting the influence of gonadal hormones, pre- and postmenopausal phase of women’s life, and sex- and age-related differences of the intake of dietary NAD^+^ precursors [53].

To evaluate the peripheral oxidative stress in depressed adolescents and adults, we quantified levels of di-Tyr and myeloperoxidase in plasma. Myeloperoxidase was previously reported as an enzyme of inflammation important in the etiology of recurrent depressive disorders [54]. Upon the activation of neutrophils in peripheral blood and tissue, MPO is released to subcellular compartments as well as extracellular environments. While chronical overproduction of MPO can cause tissue damage, it can play an anti-inflammatory role in particular situations [55]. Correspondingly, the plasmatic levels of MPO were reported higher in various diseases such as cancer [56], or in patients after myocardial infarction [57].

In our study, we observed a strong variation within our results of this marker, so we cannot draw any relevant conclusion even though there was a paradoxical trend of higher levels in controls. However, the level of MPO activity seems to be a better diagnostic tool in inflammatory conditions associated with oxidative stress than the concentration of MPO itself. To date, consensus has not been reached for standardised MPO activity measurement. Several studies documented that naturally occurring compounds such as polyphenols, melatonin, flavonoids, but also anti-inflammatory drugs show inhibitory activities against MPO [58,59]. Furthermore, niacin possesses inhibitory effects against cellular ROS production and MPO release [60]. This vitamin from the group of B vitamins was found to ameliorate depressive symptoms in animal models [61], but also in clinical studies of patients with affective disorder [62]. Moreover, this vitamin activates the HCAR2 receptor as the 3HB does [63].

Although we did not observe any relevant result from MPO level measurement, levels of possibly MPO-generated stable di-Tyr were enhanced in MDD adults compared to controls. However, we did not observe the same in adolescents with MDD. Higher levels of dityrosine might reflect higher oxidative stress and higher protein oxidation damage. Increased levels of dityrosine in blood were also found in psychiatric inpatients with previous suicide attempts and there was a positive correlation between the number of suicide attempts during a lifetime and the level of di-Tyr [64]. Since MDD is a recurrent disorder [65], levels of di-Tyr could also correlate with the number of depressive episodes, which could explain our findings; however, bigger studies are warranted to confirm this hypothesis. A short period of antidepressant therapy, as well as small sample size, were limitations to relevantly interpret the results of measured parameters in the second patient blood samples, and thus evaluate the antidepressants’ effects.

In this study, we have not confirmed the effects of antidepressants on energy metabolism and oxidative stress parameters. However, our study was limited by small sample sizes and a short period of observation. Several in vitro studies [66] and in vivo studies [67,68,69,70] have previously found an association. Not only the disorder itself but also the effect of the antidepressant could be an important topic for future studies, considering that our patients were not drug-naïve at the time of hospital admission and their medication could have affected the measured parameters. Our findings should be considered as preliminary, and larger studies could reveal if the metabolic status of MDD patients after antidepressant therapy is of high importance. A slight difference in the study design in adolescents and adults with MDD might be another limitation of our study.

## 5. Conclusions

In this study, we were able to find a significant difference in the levels of dityrosine and 3HB in adults and significantly different levels of 3HB in adolescents with MDD compared to controls. These results were obtained with high effect sizes according to power analysis. Therefore, we can conclude that the alteration of energy metabolism might be present in patients with MDD independently of age. We also confirmed increased protein oxidation damage in adult patients diagnosed with MDD. More studies are necessary to verify this in depressed children and adolescent patients and the impact of antidepressant therapy, especially after a longer duration.

## Figures and Tables

**Table 1:** Basic demographic data of depressed patients and controls.

Group		No	Age ± SD	Males/Females
Adolescents	Patients	11	16.0 ± 1.2	6/5
	Controls	20	16.9 ± 0.7	10/10
Adults	Patients	21	63.3 ± 5.8	8/13
	Controls	15	63.3 ± 6.9	12/3

**Table 2:** Comparison of levels of measured parameters between case1, case2, and controls.

Group	Parameter	CA1 ± SD	CA2 ± SD	CO ± SD	CA1 vs. CO	CA1 vs. CA2
Adolescents	DITYR	0.26 ± 0.05	0.28 ± 0.07	0.27 ± 0.03	0.4380	0.3345
	NADH	0.36 ± 0.11	0.39 ± 0.11	0.38 ± 0.05	0.2116	0.7002
	3HB	0.83 ± 0.35	0.90 ± 0.21	0.54 ± 0.09	0.0004	0.1016
	MPO	26 ± 19	25 ± 13	37 ± 31	0.5501	0.1934
Adults	DITYR	0.40 ± 0.09	0.36 ± 0.05	0.34 ± 0.02	0.0092	0.0928
	NADH	0.49 ± 0.10	0.49 ± 0.13	0.48 ± 0.06	0.6113	0.7197
	3HB	1.39 ± 0.53	1.42 ± 0.67	0.74 ± 0.13	<0.0001	0.6698
	MPO	19 ± 12	16 ± 14	48 ± 52	0.1292	0.0942

3HB—3-hydroxybutyrate, CA1—case1, CA2—case2, CO—controls, DITYR—dityrosine, MPO—myeloperoxidase, NADH—nicotinamide adenine dinucleotide. Levels of 3HB in mmol/l, MPO in ng/ml, NADH, and DITYR as ratios NADH/TRP (tryptophan) and DITYR/TRP.

## Data Availability

Data available upon request from the authors.
